# The Role of GCH1 Deficiency and Tetrahydrobiopterin in Mental Health

**DOI:** 10.3390/ijms26168030

**Published:** 2025-08-20

**Authors:** Grant E. Williams, Sharon Hausman-Cohen, Maryelaine Sotos, Emily Gutierrez, Carol Bilich, Francis W. Mueller, Shaun Jagshi

**Affiliations:** 1 IntellxxDNA^TM^, Austin, TX 78731, USA; gwilliams@intellxxdna.com (G.E.W.);; 2 Resilient Health, Austin, TX 78731, USA; 3 Neuronutrition Associates, Austin, TX 78759, USA; dremilygutierrez@icloud.com; 4 Huebner Family Medicine, San Antonio, TX 78240, USA

**Keywords:** GCH1, BH4, tetrahydrobiopterin, genomics, clinical decision support tool, precision medicine, personalized psychiatry, neurotransmitters, depression, anxiety, mental health

## Abstract

Treatment-resistant mental health concerns significantly contribute to society in terms of financial costs and individually by creating emotional and functional costs. An important yet little-recognized cause of treatment-resistant mental health conditions is tetrahydrobiopterin (BH4) deficiency. BH4 is an essential cofactor for producing serotonin, dopamine, norepinephrine, and nitric oxide—molecules critical to mood and focus. The enzyme GTP Cyclohydrolase 1 (GCH1), produced by a gene of the same name, catalyzes the first step in synthesizing BH4. Variants in this gene have been associated with low BH4 levels, as well as depression and ADHD. The case reports presented in this article illustrate that a partial BH4 deficiency, as conveyed by the *GCH1* rs841 variant, may contribute to wider issues in mental and neurological health including depression and ADHD but also severe treatment-resistant anxiety, Premenstrual Dysphoric Disorder, insomnia, complex behavioral issues, and autism. The effects of GCH1-mediated BH4 deficiency may be able to be rescued with a low-dose BH4 replacement, as illustrated by these cases, where substantial observational improvements in mental health concerns were reported in all five cases. This paper also demonstrates how a genomics clinical decision support tool can non-invasively flag “low producers” by identifying individuals with the AA genotype for *GCH1* rs841, as well as other modifiable genomic contributing factors to mental health concerns. These cases broaden the understanding of BH4′s psychiatric relevance and also serve to further the medical literature by documenting positive responses to low-dose BH4 (ranging from 0.09 to 0.3 mg/kg/day) and other genotype-guided interventions across diverse mental and neurological health presentations, highlighting the potential benefits and importance of a genomically targeted, precision approach to psychiatry.

## 1. Introduction

Tetrahydrobiopterin (BH4) is an essential cofactor for crucial brain and cellular functions, particularly the synthesis of the key neurotransmitters, serotonin (5-HT), dopamine (DA), and norepinephrine (NE) [1]. These neurotransmitters are fundamental to mental health and regulate mood, behavior, emotional responses, cognition, attention, and sleep [2,3]. BH4 also plays a vital role in nitric oxide (NO) synthesis, a molecule critical for tissue oxygenation, immune system regulation, and infection response [1]. Low-dose BH4 supplementation can significantly benefit individuals with a genetic predisposition to suboptimal yet non-lethal BH4 levels.

Studies have shown that low levels of cerebrospinal fluid (CSF) BH4 are associated with a range of neuropsychiatric and neurodevelopmental conditions, including depression, anxiety, and autism [4,5,6]. The etiology of these disorders can be linked to downstream effects on neurotransmitter levels. Additionally, Frye et al., 2010, review BH4 replacement and outcomes in individuals with autism who had low CSF BH4 levels, with significant improvements observed within 4–8 weeks at high doses [7].

**Figure 1:**
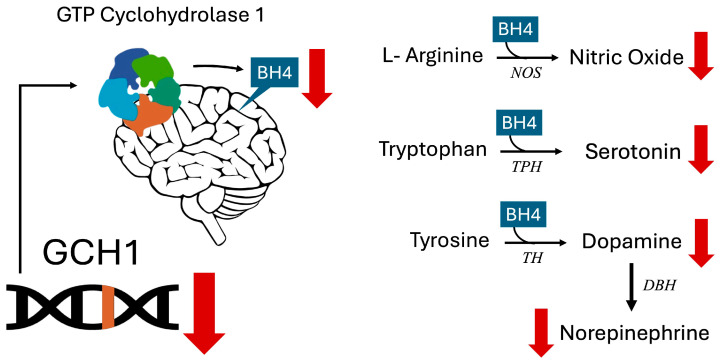
Impact of decreased GCH1 and BH4 (Tetrahydrobiopterin) on neurotransmitter synthesis. GCH1 encodes GTP Cyclohydrolase 1, the rate-limiting enzyme in BH4 synthesis. Impaired GCH1 function decreases production of the cofactor BH4. Nitric oxide synthases (NOS), Tryptophan Hydroxylase (TPH), and Tyrosine Hydroxylase (TH) are BH4-dependent enzymes involved in production of nitric oxide, serotonin, and dopamine, respectively. Dopamine is converted to norepinephrine by the Dopamine Beta Hydroxylase (DBH) enzyme. Decreased BH4 reduces production of these neurotransmitters and nitric oxide [1,4]. Black arrows with gene names symbolize enzymatic conversion by the protein product of the gene name associated with the arrow. The red downward arrows symbolize decreased production of neurotransmitter or other product.

GTP Cyclohydrolase 1 (GCH1) catalyzes the rate-limiting step in BH4 biosynthesis [1]. The rs841 single nucleotide polymorphism (SNP) in the *GCH1* gene, while not considered pathogenic, is associated with decreased gene transcription and expression, especially in homozygous individuals, leading to reduced BH4 levels [8]. BH4 is necessary for the conversion of tryptophan to 5-HT by Tryptophan Hydroxylase and for the conversion of tyrosine to DA, which is important as a neurotransmitter but also as the precursor of NE. Consequently, BH4 deficiency results in decreased levels of 5-HT, DA, and NE in the brain (Figure 1) [4,9]. The *GCH1* rs841 gene variant has been linked to various neuropsychiatric conditions, mood disorders, and behavioral traits. Homozygosity for this SNP (AA genotype) has been associated with ADHD traits including impaired sustained attention and vigilance [10]. Furthermore, the A allele is more prevalent in depressed patients unresponsive to Selective Serotonin Reuptake Inhibitors (SSRIs) (suggesting lower serotonin levels) [11] and in individuals with lower novelty-seeking scores (indicating reduced dopamine levels) [12].

This paper will expand this evidence by providing multiple additional examples of individuals with BH4 deficiency due to *GCH1* rs841, including those with treatment-resistant anxiety, severe behavioral issues, Attention-Deficit/Hyperactivity Disorder (ADHD), Premenstrual Dysphoric Disorder (PMDD), and Autism Spectrum Disorder (ASD). Additionally, these case reports from multiple sites will take the literature one step further by showing that low-dose BH4 replacement can significantly improve outcomes for individuals with two copies of *GCH1* rs841. Patients of a variety of ages are discussed, and their effective dosing is reported to provide a framework for clinicians as to how to potentially address this important, possibly reversible cause of treatment-resistant mental health disorders.

Genomically targeted interventions in mental health are well-established for variants such as Methylenetetrahydrofolate Reductase *(MTHFR) C677T*, where L-methylfolate is widely available and effective for depression and mild cognitive impairment [13,14]. BH4 replacement can be as powerful, if not more powerful, as a genomically targeted intervention for the approximately 4% of individuals with low-functioning, but non-pathogenic *GCH1* variants. Non-genomically targeted studies of BH4 replacement are unlikely to accurately assess efficacy due to the low prevalence of *GCH1* rs841 homozygosity, a genetic predisposition to low BH4. Therefore, further research and targeted trials focusing on genomics and precision medicine, including *GCH1*/BH4 and other relevant variants, are essential to optimize outcomes for treatment-resistant mental health disorders.

## 2. Results

### 2.1. Case 1: Patient A

#### 2.1.1. Patient Background

Patient A, an 11-year-old female, presented with a chief complaint of severe anxiety that has persisted since early childhood. Her anxiety was debilitating, leading to significant periods of homebound schooling each year beginning in the second grade. The patient had seen many different practitioners over the years including child psychiatrists, therapists, and functional nutritionists. Despite trials of multiple medications over the past 3 years including fluoxetine and other various types of psychotherapy, along with consultations with both integrative clinicians and nutritionists, her symptoms remained resistant to treatment. She was on 40 mg of fluoxetine at the time of presentation, but her anxiety was still severe, and her parents did not feel that the fluoxetine was helping. The hope of the parents was that with genomic insight and targeted interventions, she would be able to receive improvements in her symptoms and wean off the fluoxetine.

Of note in Patient A’s history is the exacerbation of her anxiety during periods of illness, such as when she contracted viruses (COVID-19 specifically, but not exclusively). To gain an understanding of what was contributing to her issues and symptoms, the IntellxxDNA™ (IXXD) Mental Wellness genomics report was ordered by her physician.

#### 2.1.2. Genomic Results and Interpretation

A genomic analysis revealed several variants that helped explain Patient A’s persistent symptoms of severe anxiety and poor response to previous treatments:

- *GCH1* (GTP Cyclohydrolase 1) rs841 homozygous: This SNP is associated with a lower GCH1 expression and BH4 production, which in turn impairs serotonin production and contributes to anxiety [4,8,9]. The rs841 is also associated with a decreased response to SSRIs [11], an issue this patient was experiencing with fluoxetine. Given that infections deplete nitric oxide and increase NO requirements [15], and that BH4 is a cofactor required for NO production [1], the BH4 impairments seen with this SNP shed light on why she became markedly pale with exacerbated anxiety symptoms when ill.
- *GSTO1* (Glutathione S-Transferase Omega 1) homozygous: GSTO1 is needed for the recycling of vitamin C [16], a cofactor required for norepinephrine synthesis [17], and variants have been associated with a lower GSTO expression in the brain of autopsied subjects [16]. Impaired vitamin C recycling, along with insufficient BH4 due her lower functioning GCH1, likely resulted in further reductions in norepinephrine that impacted the patient’s mood [18,19,20].
- *DRD2* (Dopamine Receptor D2) homozygous: Patient A’s DRD2 SNP is associated with decreased dopamine signaling [21,22]. Her low BH4 likely further exacerbated her dopamine deficiency (impacting pleasure and motivation), as it is a cofactor required for synthesis [1].
- *HTR1A* (5-Hydroxytryptamine Receptor 1A) homozygous: Patient had two copies of an overactive HTR1A serotonin autoreceptor that contributed to reduced serotonin neurotransmission [23]. It is associated with 2.8x the risk of comorbid generalized anxiety disorder and depression [23].

#### 2.1.3. Intervention and Post-Treatment Improvements

Patient A’s treatment regimen most importantly included adding a low dose compounded prescription of sapropterin (BH4), initially dosed at 2.5 mg twice daily to address her *GCH1* variants. Her parents were given instructions on titrating up if they did not observe improvements in anxiety symptoms and mood and if no side effects were reported with the initial BH4 dose. After 6 months, her optimal BH4 dose was determined to be 5 mg twice daily with adjustments (temporary increases) made during episodes of the flu, COVID-19 and other viruses due to increased BH4 and NO production needs.

In addition to BH4, her provider also recommended supplements to further support serotonin and gamma-aminobutyric acid (GABA, a calming neurotransmitter) synthesis and signaling. This included a supplement blend of 100 mg 5-HTP, 23 mg zinc, 30 mg saffron, and B vitamins (10 mg riboflavin, 10 mg B6, and 1000 mcg B12), along with 5000 mcg methylfolate and 2500 mcg folinic acid. Vitamin C was recommended to address her *GSTO1* variants. She also started N-acetylcysteine (NAC) to manage obsessive–compulsive disorder (OCD) tendencies (skin picking) [24] but discontinued usage after a relatively short period of time. Regular exercise was encouraged to manage adrenaline levels and boost brain-derived neurotrophic factors (supporting neuronal health and mood) [25].

After a few months of this personalized plan, Patient A experienced significant improvements. She began participating in clubs, going to sleepovers, and attending social events with friends for the first time. At the time of her follow-up, she had not missed a single day of school due to anxiety, which was drastically different than previous years. The parents reported being “over the moon happy” with the positive changes, as their daughter was finally able to enjoy a normal teenage life. After approximately 6 months of her treatment regimen, she was able to decrease and stop all supplements with the exception of BH4. Anxiety continued to be fully controlled after 6 additional months of being off the extra support. At this point, she started to taper down on her fluoxetine from 40 mg to 20 mg. The patient did initially experience temporary setbacks during times of illness. During those times, she resumed the NAC, vitamin C, and supplement blend and increased BH4 to 7.5 mg twice/day. Approximately 1.5 years after starting this approach, she was able to completely taper off the fluoxetine and subjectively reports having zero anxiety. Unless she is ill, Patient A takes no other supplements.

While lab values are not generally followed in the mental health world, Patient A’s were of interest. Her baseline findings revealed significantly elevated fasting insulin levels of 37. Even though she was only 12 years old, she was prediabetic with an HgA1c of 5.9. A search of the literature shows that BH4 is essential not only for the production of mood-related neurotransmitters but also for suppressing hepatic gluconeogenesis [26]. Administration of BH4 to diabetic mice lowered fasting blood glucose and had beneficial effects on glucose intolerance as well as insulin resistance. These effects were largely attributable to the BH4-dependent endothelial nitric oxide synthase (eNOS) pathways (eNOS dysfunction induces glucose intolerance and insulin resistance) [9,26]. After 3 months of BH4, her fasting insulin levels dropped over 50% from 37 to 15, and her HgA1c was reduced to 5.6.

### 2.2. Case 2: Patient B

#### 2.2.1. Patient Background

Patient B, a 42-year-old female, presented with a chief complaint of severe PMDD, which worsened after the birth of her second child. Her treatment history included oral contraceptive pills (OCPs), which gave partial relief. IXXD’s Mental Wellness genomic report was ordered to gain a deeper understanding of what might be contributing to her symptoms.

#### 2.2.2. Genomic Results and Interpretation

A genomic analysis revealed several key variants contributing to her severe PMDD:

- *GCH1* (GTP Cyclohydrolase 1) rs841 homozygous: Given BH4′s role in serotonin production [1], and the importance of serotonin in the alleviation of PMDD [27], this variant was believed to be a potential strong contributor to her severe PMDD.
- *FOLR1* (Folate Receptor Alpha, FR-α) heterozygous: Patient B had a rare *FOLR1* variant that added strain to her dysregulated neurotransmitter production triggered by GCH1. This variant is known to decrease FR-α transport capabilities, creating a relative cerebral folate deficiency when methylfolate is used as the folate source [28,29].
- *MTHFR* (Methylenetetrahydrofolate Reductase) C677T homozygous: Associated with an approximate 70% reduction in enzymatic activity, MTHFR converts folic acid into methylfolate, which is the form of folate that is most able to cross the blood–brain barrier. Methylfolate or a bioactive folate that can enter the brain is crucial for making serotonin, norepinephrine, and dopamine in the brain [30,31,32]. The combination of *FOLR1* [33] and the *MTHFR* variants [34] could compound the problems caused by a *GCH1*-induced BH4 deficiency and have additive effects on downstream neurotransmitters [1].
- *ESR2* (Estrogen Receptor Beta) homozygous: Decreased ESR2 signaling seen with this SNP. This variant has been associated with mood disorders during times of low estrogen exposure, including anxiety [35] and severe depression among postmenopausal women not on hormone therapy [36]. This variant, combined with the natural drop in estrogen that occurs before menstruation [37], helped explain why her mood symptoms peaked during that part of her cycle.
- *IL10* (Interleukin 10) homozygous: Patient B’s well-studied *IL10*-lowering SNP may have further contributed to her inflammation and premenstrual symptoms, as IL10 is anti-inflammatory [38]. ESR2 also normally modulates inflammation via IL10, and thus the combination of low ESR2 signaling and low IL10 may compound each other’s effects [39].

#### 2.2.3. Intervention and Post-Treatment Improvements

Patient B was initially put on BH4 2.5 mg twice per day to address *GCH1*, with the option to take a third dose of 2.5 mg when mood symptoms were severe. Normally, for *MTHFR* variants, methylfolate, which has been well studied in the literature and shown to help with treatment-resistant depression [40], would be added. Given her combination of *MTHFR* with *FOLR1*, however, a different approach was taken. For her low-functioning *FOLR1* variant, supplementing with folate in the form of folinic acid, which can cross into the brain using the alternative reduced folate carrier [30], was deemed medically necessary to support adequate brain serotonin synthesis. Patient B’s physician added the previously mentioned supplement blend that provides adequate amounts of folinic acid as well as methylfolate and other cofactors for serotonin and neurotransmitter synthesis.

To address *ESR2* and *IL10*, her physician stopped her OCPs and instead started low-dose hormone replacement therapy. This consisted of an estrogen patch to provide a more stable dose via the transdermal route along with a compounded testosterone cream. To provide additional anti-inflammatory support, the patient began an omega-3 supplement at a dose of 2 g daily, as it has been shown to increase IL10 levels [41]. Within a few weeks of making these changes, she noted an improvement in all symptoms, most notably mood lability. She has continued with this regimen to date. Patient B did temporarily stop taking the supplement blend but noted feeling increased lability in her mood during those 3 weeks and thus resumed it. Approximately 22 months later, the patient reported she is still doing well and managing her PMDD symptoms with a dose of 2.5 mg BH4 2-3x daily. This case highlights how interacting genomic pathways can lead to more pronounced effects.

#### 2.2.4. Case 2.2: Patient B2, Patient B’s Son

Patient B’s 9-year-old son, Patient B2, also underwent genomic testing due to concerns around his ADHD behaviors and difficulty transitioning from one activity to another. Both he and his mother shared several SNPs associated with mood disorders, including GCH1 rs841 homozygosity, which has been associated with ADHD traits including impaired sustained attention and vigilance [10]. He also started BH4, but at a lower dose of 2.5 mg daily. Additionally, Patient B2 was recommended to start transdermal glutathione and encouraged to increase vitamin C containing foods to support the conversion of dopamine to norepinephrine [42]. He did stay on the BH4 but did not continue with the glutathione and vitamin C support. His mother reported improvements in ADHD behaviors, and that he continues to do well with 2.5 mg BH4 daily and therefore did not need to titrate up on the dose.

### 2.3. Case 3: Patient C

#### 2.3.1. Patient Background

Patient C, a 10-year-old male, presented with high-functioning ASD (level 1) and ADHD. His mother described his mood as irritable, explosive, and impulsive. Patient C reported as having failed multiple medications for attention and mood since kindergarten, as prescribed by his neurologist, including stimulants and SSRIs. His Autism Treatment Evaluation Checklist (ATEC) score was 36 at treatment onset, which is consistent with mild ASD (the average ATEC score for a neurotypical child of the same age would be 22) [43]. To gain a clearer understanding of the contributing factors to his ASD and ADHD, IXXD’s neurodevelopmental genomics report that focuses on modifiable gene variants associated with ASD, ADHD, anxiety, OCD, and other neurobehavioral issues was ordered.

#### 2.3.2. Genomic Results and Interpretation

A genomic analysis revealed multiple clinically significant variants that contribute to ASD, ADHD, and behavioral issues:

- *GCH1* (GTP Cyclohydrolase 1) rs841 homozygous: Just like the cases above, Patient C had two copies of this SNP that is associated with a lower GCH1 expression and BH4 production, impairing his ability to synthesize neurotransmitters [8,9].
- *MTHFR* (Methylenetetrahydrofolate Reductase) C677T homozygous: As discussed above, methylfolate is the form of folate that most easily crosses the blood–brain barrier and is a critical cofactor, along with BH4, for synthesizing serotonin from tryptophan, dopamine from tyrosine, and norepinephrine from dopamine [1,32]. This *MTHFR* variant is a strong contributing factor to ASD [31]. Additionally, *MTHFR C677T* can contribute to an increased risk of developing folate receptor antibodies [44], which this patient had.
- *PPCDC* (Phosphopantothenoylcysteine Decarboxylase) homozygous: This particular *PPCDC* variant Patient C had two copies of is associated with low zinc [45]. Low zinc is known to contribute to ASD [46].
- *DBH* (Dopamine Beta Hydroxylase) homozygous: DBH is the enzyme that converts dopamine to norepinephrine. Low functioning DBH variants are significant contributors to ADHD [47]. Increased dopamine and decreased norepinephrine have also been associated with irritability and increased ASD severity [48].
- *HTR1B* (5-Hydroxytryptamine Receptor 1B) homozygous: Variants in the HTR1B serotonin receptor pathway contributed to impaired serotonin signaling and are associated with reduced impulse control and inattentive traits of ADHD [49,50].

#### 2.3.3. Intervention and Post-Treatment Improvements

Prior to starting BH4, treatment with a custom compounded nutrient formula was started to address some of the above key contributing genomics pathways, although the mother admits there have been difficulties in getting Patient C to take vitamins and medications, so this compound was given more sporadically. This compound included: vitamin C (200 mg/day), a cofactor for DBH enzyme and norepinephrine synthesis [42], zinc (10 mg/day) to address the PPCDC pathway, reduced forms of folate (both folinic acid, which has benefit for addressing folate receptor antibodies and methylfolate to address MTHFR), as well as the cofactors needed for MTHFR and the methylation cycle such as B12, pyridoxal 5′-phosphate (P5P), and riboflavin. Riboflavin is particularly important for MTHFR C677T homozygous individuals as was highlighted in the genomics clinical decision support tool, because this variant is in the riboflavin binding site, and improved outcomes have been demonstrated in these individuals when both riboflavin and methylfolate are supported [51]. Vitamin D (4000 IU/day) [52] and NAC (600 mg/day) [53] were also included to further support ASD and ADHD treatment and Patient C’s genomics. A compounded custom mixture was chosen in an effort to increase patient compliance and reduce pill burden, but again, it was taken with poor compliance due to difficulties with taste and tolerability, which is common with young children.

Then, Patient C’s mother later added in the BH4 when there was no other variable. Compounded prescription BH4 was started at a low dose of 2.5 mg every morning for 1 month to address GCH1. The patient was then moved up to 2.5 mg twice a day, as there was not a noticeable response on the once daily formulation. After 2 months of BH4 at 2.5 mg twice daily, his mother reported significant progress, although there was still room for improvement. His mother reported “Overall, he seems less explosive. He regulates after getting angry much quicker. His attention during language intense activities is still weak. His stimming fluctuates a lot. However, he’s now sitting and doing his math homework independently which he could not focus on his own 3 months ago.” The BH4 dose was further increased after 3 months to 5 mg of BH4 in the morning and 2.5 mg at night. After moving up to this higher dose, Patient C started having some high-dopamine-type symptoms. Rather than decreasing the dose back to the dose that resulted in the initial behavioral improvement, the patient was advised by a different physician participating in his care to do a trial of stopping the BH4 and compounded supplements. The physician felt uncomfortable with the off-label usage of medications and supplements and was not familiar with the field of mental health, ASD related genomics, or genomics being used for clinical decision support. The other variable was that the pediatrician created worry amongst the parents by stating that there was not enough evidence for the supplements and BH4 for this patient. The mother and father were weary of any pharmaceuticals to begin with, so again, they stopped the BH4. Patient C regressed and became more irritable when off the BH4. Symptoms did not improve until his mother later added back the BH4 2.5 mg twice daily, and behavior again improved. Patient C’s mother is pleased and optimistic with the behavioral gains and gains in attention and focus, which are being noted. More recently, low-dose amantadine, which is a dopamine reuptake inhibitor, was added to further increase dopamine levels in the brain to help with residual irritability, and the family reports he is now doing well.

### 2.4. Case 4: Patient D

#### 2.4.1. Patient Background

Patient D is a 57-year-old woman with a lifelong history of depression, anxiety, and chronic insomnia. She has tried multiple medications over the years without achieving full symptom relief. Bupropion was ineffective, and although escitalopram has helped manage her depression, she continues to experience intermittent anxiety and persistent sleep disturbances, even with the use of benzodiazepines.

#### 2.4.2. Genomic Results and Interpretation

A genomic analysis revealed several variants that may explain her persistent symptoms and partial response to treatment:

- *GCH1* (GTP Cyclohydrolase 1) homozygous: This low-functioning SNP suggests a reduced production of tetrahydrobiopterin [8], a cofactor required for the synthesis of serotonin, dopamine, and nitric oxide [1].
- *GC* (Vitamin D Binding Protein) homozygous: Patient D carries two copies of a variant strongly associated with low vitamin D levels and impaired transport of active vitamin D metabolites [54,55]. Low vitamin D may have worsened her depression, as vitamin D is essential for serotonin synthesis [56] and is linked to both depression and anxiety [57].
- *HTR2A* (5-Hydroxytryptamine Receptor 2A) homozygous: Impairs serotonin receptor function and is associated with reduced serotonin signaling, decreased GABA activity, and increased cortisol levels—all of which are relevant to anxiety and sleep disturbances [58,59].
- *GSTO1* and *GSTO2* (Glutathione S-Transferase Omega 1 and 2) haplotype: These enzymes support detox pathways, and the variants are associated with decreased enzyme activity and a reduction in the body’s ability to recycle vitamin C [16,60], which is crucial for norepinephrine synthesis—a neurotransmitter important for energy and concentration [17].

#### 2.4.3. Intervention and Post-Treatment Improvements

Patient D was started on BH4 at 5 mg daily, with encouragement to gradually increase the dose based on her response. She eventually titrated up to 7–10 mg twice daily, reporting significant improvements in her mental health within four weeks of reaching the higher dose.

In addition to BH4, based on her genomics, Patient D started taking L-theanine and herbal GABA mimetics (valerian, chamomile, and passionflower) as needed to help with sleep and decrease the frequency of the prescription benzodiazepine she had been using intermittently for sleep. Additionally, based on her genomics, vitamin D (5000 IUs), which increases serotonin synthesis [56], and vitamin C (500 mg/day), to help with norepinephrine synthesis [17], were integrated into her supplement regimen at the same time the BH4 treatment was started. Patient D’s mental health and overall health have significantly improved with her BH4 and genomically targeted plan, although she had concerns regarding the high cost of BH4.

She described a feeling of general wellness for the first time in years. Unlike previous medications, BH4 did not make her feel sedated or drugged; instead, she reported feeling normal and clear-headed. Her anxiety episodes became less frequent, and, most notably, her sleep improved dramatically. Prior to BH4, she described waking unrefreshed but now reports waking spontaneously at 6 a.m. feeling rested and ready to start her day—an experience she had never previously enjoyed.

This case underscores the clinical relevance of genomics in long-standing mental health conditions. Variants in GCH1, vitamin D metabolism, HTR2A (serotonin regulation), and detoxification pathways contributed to Patient D’s persistent symptoms. Targeted intervention with BH4 led to a marked improvement in mood, anxiety, and sleep—achievements not reached with conventional pharmacotherapy alone.

### 2.5. Case 5: Patient E

#### 2.5.1. Patient Background

Patient E, an 8-year-old boy, was meeting early developmental milestones and was neurotypical as a baby. At 18 months, he started developing self-injurious behavior in that he began headbanging to fall asleep. By age 4, he displayed severe emotional outbursts and self-harm threats, such as saying he would jump out of a window. He started psychotherapy at that time. At age 7.5, following a growth spurt, symptoms worsened: increased aggression, tantrums, and intense nighttime outbursts, often followed by calm behavior.

When his brother was hospitalized for typhus, Patient E was tested (including CBC, chemistry, EBV, CMV, and cultures)—all was normal, except for an elevated CRP and ESR. Guanfacine (which is known to lower norepinephrine [61]) worsened symptoms and was stopped. A psychiatric evaluation diagnosed him with generalized anxiety disorder (with OCD traits), ADHD (combined), and Major Depressive Disorder. Trials of risperidone and aripiprazole worsened his agitation and caused twitchiness. He was also started on sertraline and methylphenidate.

#### 2.5.2. Genomic Results and Interpretation

- *GCH1* (GTP Cyclohydrolase 1) homozygous: Low-functioning genotype that would have led to lower serotonin, norepinephrine, dopamine, and nitric oxide [4,9]. Infections would serve to further deplete BH4 and exacerbate symptoms [1].
- *CCL2* (C-C Motif Chemokine Ligand 2) homozygous: Patient E had two copies of a SNP found in roughly 9% of the population that is associated with increased CCL2 [62]. Increased activity of this chemotactic factor can translate into more brain inflammation and tissue destruction at the area of inflammation [63].
- *IL1B* (Interleukin 1 Beta) homozygous: A heightened inflammatory response to infections and other. Highly associated with ASD and other neurobehavioral issues [64,65].
- CUBN (Cubilin) homozygous: Low B12 absorption in gut due to lower intrinsic factor, thus increasing B12 needs [66].
- MTRR (Methionine Synthase Reductase) homozygous: Increased need for B vitamins, also a significant contributor to ADHD [67].
- HTR1A (5-Hydroxytryptamine Receptor 1A) homozygous: Low serotonin synthesis due to serotonin dysregulation [68].
- GSTP1 (Glutathione S-Transferase Pi 1) homozygous: Reduced detox capacity and less ability to process and remove glyphosates [69].

#### 2.5.3. Intervention and Post-Treatment Improvements

Targeted supplements were recommended, including BH4 2.5 mg daily to start (increased to twice a day after a few days), along with palmitoylethanolamide, resveratrol, and a mood supporting product with 5HTP and B vitamins to help address some of the key genomics above. Patient E’s parents wanted to only take one thing at a time, so only the BH4 was started.

Initial improvement was noted during the first few weeks after starting the BH4. After a month on the supplement, Patient E had an outburst with worsening behavior and twitchiness. This outburst was eventually determined to likely be due to the addition of aripiprazole, an atypical antidepressant/antipsychotic prescribed by the child’s psychiatrist. Aripiprazole can act as both a dopamine antagonist and dopamine agonist depending on dopamine levels and other conditions [70]. Aripiprazole, given in combination with BH4, likely led to an elevation in dopamine. Stopping the aripiprazole resolved the issue. Once the child was off the aripiprazole, the mother was able to increase the BH4, which led to notable improvements. The parents, at one point, ran out of the medication, which further allowed them to see the benefits it was conveying. After a lapse in BH4 for a few days, Patient E quickly had a return of symptoms including outbursts and unpredictable behavior. Within 1 day of receiving the BH4 medication (sapropterin), he returned to a calm state. The mother is very excited with this positive response. She is now considering trying some of the other genomically targeted supplements to see if her son can make further gains.

Both this case, where the mother never started the other supplements, and Case 1 (Patient A), where the child stopped everything but the BH4, highlight the value of genomic insights in guiding treatment, especially in complex pediatric cases. Coordinated care among providers and creative strategies for administering supplements are essential to achieving effective outcomes.

A summary table of each case report patient and their respective BH4 doses used in ongoing treatment plans is presented below (Table 1). Note that in these cases, as previously discussed, clinicians started with a very low BH4 dose, which would generally be considered an “otc” supplement dose rather than a prescription dose, and gradually increased if needed. Increases were based on the extent of patient or parental reported observational improvements in symptomology (or lack thereof) and the absence of side effects.

## 3. Discussion

The molecular mechanisms behind mental health, mood, and neurodevelopmental disorders such as depression, anxiety, ADHD, and ASD are typically diverse and complex. Dysregulated neurotransmitter pathways are often at the crux. This dysregulation can stem from genomic variants that lead to lower levels or activity of neurotransmitter-supporting cofactors, enzymes, regulators, and precursors [71,72]. Beyond the established links to Major Depressive Disorder [11] and ADHD traits [10], these case studies illustrate that a genomically induced GCH1 deficiency may be a significant contributing factor to a range of mental health conditions including treatment-resistant anxiety, PMDD, ASD, and other behavioral issues. Each of the case study patients were homozygous for GCH1 rs841 (AA genotype), which is associated with a reduced GCH1 expression and enzyme activity and lower production of BH4 [8,9]. Low BH4 production conveyed by the GCH1 variant decreases the ability to synthesize serotonin, dopamine, norepinephrine, and nitric oxide, as BH4 is a necessary cofactor [1,4].

In the literature, BH4 replacement has been shown to have benefits in individuals with low CSF BH4 and ASD [7], as well as, of course, for Phenylketonuria (PKU) [73]. Prior studies of BH4 supplementation in non-PKU patients are largely restricted to ASD across a variety of observational, open-label, double-blind, placebo-controlled trials. The doses in these ASD studies range from 1 to 20 mg/kg BH4 per day, with the higher dose of 20 mg/kg day showing the most consistent improvements in primary outcomes such as social awareness, communication, and behavior. Adverse effects such as irritability and GI issues were generally noted to be minimal and mild [5,7]. Regarding depression, the evidence in existing smaller studies is mixed and limited with contrasting BH4 treatment results [1]. For ADHD and anxiety, human trial data is lacking connections that are largely restricted to mechanistic links and animal models [4,5,74]. Results from these case studies indicate that individuals with mild to moderate reductions in BH4 conveyed by GCH1 rs841 can be identified with genomics and may significantly benefit from low-dose replacement. The doses used in this report range from 0.088 to 0.292 mg/kg per day due to variability in factors such as age, primary concerns, and the severity of issues. These doses, however, are a magnitude of order lower than what was used in ASD and other relevant studies. The beneficial effects of these lower doses may span across the broader field of mental health, as illustrated by the patient cases above. 

Some clinically significant genomic contributors and their respective pathways and genomically targeted therapies are well-documented. For example, there are over 8400 publications discussing *MTHFR C677T* (rs1801133) in the NCBI database and over 200 publications relating to the clinical utility of methylfolate. In comparison, there is a paucity of the literature discussing the role of less common *GCH1* variants and brain health, with only 650 total publications relating to the *GCH1* gene and 21 publications that mention the lower-functioning *GCH1* rs841. This is likely in part due to the prevalence of the common low-functioning *MTHFR C677T*, and the earlier discovery and classification of clinical impact. Based on NCBI’s ALFA minor allele frequencies, 11.3% of the population is homozygous for *MTHFR C677T*. In comparison, *GCH1* rs841 is found in the homozygous form in only 4.1% of the population. Given the sizable percentage of the population that has *MTHFR C677T*, more individuals in initial L-methylfolate studies derived benefit [75]. This in turn prompted additional, larger clinical trials that further showcased L-methylfolate’s efficacy for use in depression and other neuropsychiatric disorders [76]. Years later, methylfolate is now widely recognized by physicians as an intervention to consider for depression and other mental health concerns, and articles discussing dosing for these indications are easily accessible in the published literature. This is not the case with tetrahydrobiopterin.

To date, few clinicians are familiar with the functions of BH4 and even fewer would consider prescribing it for mental health conditions. Most think of BH4 or sapropterin as an orphan drug, with PKU as its sole indication [77]. Prescription doses designed for PKU patients are approximately 10 to 50-fold higher than what appears to benefit those with mild BH4 deficiencies based on case reports and clinician experience, thereby making it difficult to treat mild BH4 deficiency and the resulting mental health concerns without the custom compounding of BH4 or sapropterin. Off-label use can also be cost-prohibitive as it is not covered by insurance, with a typical low-dose compounded prescription costing a patient anywhere from USD 150–300 a month. In addition to difficulties with accessibility and the lack of published dosing guidelines, identifying non-PKU individuals that would benefit from BH4 replacement has historically been challenging. This is in part due to differential tissue expression and BH4 blood levels not being indicative of brain levels. Cavaleri et al., 2023, found that levels of BH4 in the blood did not differ between depressed patients and controls [78], but the postmortem brains of individuals with severe depression had a low CSF and brain levels of BH4 [4]. For this reason, confirming BH4 deficiency has traditionally required invasive CSF sampling [7]. A precision genomics approach, however, can help overcome and ultimately eliminate these limitations. The case studies presented above highlight the benefits of genomically supported personalized medicine and can provide initial guidance regarding the lower typical starting doses of BH4 for individuals with GCH1 deficiencies while research in this field continues to progress.

When used in the format of a well-referenced clinical decision support tool, genomics enables the interrogation of non-pathogenic but disease-contributing variants, a precision treatment approach without invasive procedures, and improved clinical outcomes by addressing the root causes of various conditions. This approach is particularly important for brain science and mental health due to an inability to accurately measure brain markers in the blood due to the blood–brain barrier. When a disease is labeled “treatment resistant” by physicians, this often means the root cause for that specific patient has not been identified and is not being addressed. Although the primary focus of this paper is on one particular non-pathogenic SNP (the low functioning GCH1 rs841 contributing to low BH4 and various psychiatric concerns), there are virtually always other contributing variants. By switching to an N-of-1 approach, patient outcomes have been shown to improve, sometimes with mild benefits and sometimes with dramatic improvement. Early evidence of the ability for genomically targeted treatments to reduce trial and error and improve patient outcomes has already been demonstrated in regard to dementia [79], ASD [80], and weight management [81]. Additionally, cognition-enhancing interventions in aging have been shown to rely on genomic and neurochemical modulation, reinforcing the value of BH4-related approaches in neuropsychiatric resilience (Milic et al., 2021) [82]. However, there is a need to further expand the use of and research regarding clinical decision support tools, which can improve outcomes and the mental health and lives of millions. Ultimately, now that precision medicine has become a reality, the standard of care needs to shift towards this approach. Currently, even with pharmacogenomics, less than 40% of treatment-resistant patients with depression achieve remission [83]. Going deeper and looking at the broader root causes of depression and other mental health concerns can benefit millions. As the cost of genomics has become more affordable, and more tools are being developed to help clinicians interpret and respond to an individual’s genomics, many of the previous barriers preventing personalized medicine are being reduced. Treatment-resistant depression and other mental health concerns create a multibillion dollar a year economic burden. Being able to help even 10% of individuals with treatment-resistant depression, anxiety, ADHD, and other mental health concerns would save the health care systems billions of dollars a year [84,85].

Limitations of this study include the fact that genomic contributors to neuropsychiatric conditions beyond the GCH1/BH4 pathway were addressed simultaneously, making it difficult to verify or isolate the effects of the low dose BH4 treatment, although several case study patients stopped or chose not to use the non-BH4 interventions. Additionally, a limited number of cases are included in this report and each patient served as their own historic control rather than a formal, randomized controlled trial. Validated metrics, measurements, and statistical analyses are lacking, and the extent of improvements were often observational and subjective. No standardized rating scales were dictated as these are retrospective case reports, and improvement and outcome data across the various disorders are subjective. Additionally, patient compliance and consistency in adhering to treatment regimens are limitations. Future genomically targeted research on a larger scale, including randomized and placebo-controlled trials, is warranted for specific mental health and mood disorders. These trials will provide benefits for evaluating long-term effects with follow-ups to confirm the sustainability of clinical benefits, while enabling further refinement of the ideal dosing and evaluation of potential adverse effects of lower-dose BH4 treatment. Further prospective research with robust statistical analysis would make these results more generalizable to the full population of individuals with two copies of GCH1 rs841.

## 4. Materials and Methods

The patients in this case study report are individual cases who presented to their respective clinicians, spread throughout Texas, with a variety of previously diagnosed mental health and other neuropsychiatric concerns. Due to the complexity or treatment-resistant nature of each case, clinicians recommended genetic testing for their patients to gain a deeper understanding of the root causes and how they can integrate a personalized treatment plan with standard treatments. Consent was obtained from each patient or their parent prior to purchase and sample submission. DNA samples were collected via buccal swabs by the patients at their primary residence and sent via mail to a CLIA/CAP certified lab in Houston, TX, USA for analysis on a custom Next Generation Sequencing panel with probes supplied by Twist Bioscience in San Francisco, CA, USA. Specimens were processed on the Illumina (San Diego, CA, USA) NovaSeq 6000 instrument, and genomic results were provided to physicians via the proprietary and commercially available IntellxxDNA™ (Austin, TX, USA) Clinical Decision Support Tool (CDS). The average coverage of the panel was approximately 250 reads per targeted region. To ensure accurate and reliable genotyping data, the minimum coverage required for GCH1 rs841 and other high-impact genomic variants was set at 100 reads per targeted region. Failure to achieve this coverage threshold resulted in sample reprocessing or submission and subsequent analysis of a new DNA specimen. The current recommended minimum number of reads or coverage depth for NGS in clinical care or pharmacogenomics is 30–50 reads per targeted region, so the CDS is well above the generally accepted quality threshold. Genotype calling of the CDS was also validated using known “Genome in a Bottle” samples and reference materials from the Coriell Institute, Camden, NJ, USA. Validation results showed the sequencing platform performed with greater than 99.9% accuracy.

Curated genomic information in IXXD reports was presented to the patients’ ordering provider with several sections for SNP: discussions on genes and the impact of variants on gene expression or protein function, clinical significance and associations with medical conditions or molecular pathways, and potential intervention strategies in the form of supplements, food, nutrients, medications, or lifestyle changes that mechanistically address each pathway. All information provided in the CDS was non-diagnostic, referenced, and based on the peer-reviewed literature. While a variety of potential intervention options were conveyed for each SNP, the ordering clinician made the treatment decision to utilize BH4 when 2 low-functioning *GCH1* variants were present, along with other therapeutic modalities when deemed appropriate. Patients (or parents) were informed by their clinicians that BH4 would be an off-label treatment to be integrated with current conventional treatments when applicable. Verbal consent was obtained by patients (or parents) prior to the initiation of BH4 treatment.

The extent of post-genomic treatment improvement was assessed using a variety of methods but was largely restricted to patient or parental reported behavioral and mood improvements related to the given disorder (s), as well as observational life achievements and milestones. Given the nature of this being a group case study report presented for proof of concept, there were no specified controls, randomization, or blinding. Instead, each patient served as their own historical control since they had tried and failed multiple regimens. Inclusion criteria for cases included if the patient had an ongoing mental health or neuropsychiatric concern, had tried multiple previous medications or therapies with no or minimal reported improvements in symptoms, had the AA genotype of *GCH1* rs841, and was willing to start BH4 treatment. Consent for inclusion as a case study in this publication was obtained via patient or parental approval via a signed consent form.

## Figures and Tables

**Table 1:** Final BH4 doses used in each case study.

Case Study Patient	Age, Sex, Weight, and Primary Concern(s)	GCH1 rs841 Genotype	BH4 Dose	BH4 Dose by Weight
Patient A	11, Female, 102 lbs, and Severe Anxiety	AA	5 mg BID	0.216 mg/kg/day
Patient B	42, Female, 125 lbs, and PMDD	AA	2.5 mg BID-TID	0.088–0.132 mg/kg/day
Patient B2	9, Male, 52 lbs, and Mild ADHD Behaviors	AA	2.5 mg QD	0.106 mg/kg/day
Patient C	10, Male, 66 lbs, ASD (Level 1), and ADHD	AA	2.5 mg BID	0.167 mg/kg/day
Patient D	57, Female, 151 lbs, Depression, Anxiety, and Chronic Insomnia	AA	7–10 mg BID	0.204–0.292 mg/kg/day
Patient E	8, Male, 55 lbs, and Complex Behavioral Issues (Diagnosed Anxiety with OCD traits, ADHD, and Major Depressive Disorder)	AA	2.5 mg BID	0.2 mg/kg/day

QD = once daily; BID = twice daily; TID = three times daily; and lbs = weight in pounds.

## Data Availability

Relevant genomics and data presented in paper. Full access to genomics is part of an online resource available to ordering clinicians and is not available in downloadable or printable form.

## Abbreviations

The following abbreviations are used in this manuscript:

BH4	Tetrahydrobiopterin
5-HT	Serotonin
DA	Dopamine
NE	Norepinephrine
NO	Nitric Oxide
CSF	Cerebrospinal Fluid
GCH1	GTP Cyclohydrolase 1
SNP	Single Nucleotide Polymorphism
SSRIs	Selective Serotonin Reuptake Inhibitors
ADHD	Attention-Deficit/Hyperactivity Disorder
PMDD	Premenstrual Dysphoric Disorder
ASD	Autism Spectrum Disorder
MTHFR	Methylenetetrahydrofolate Reductase
IXXD	IntellxxDNA™
CDS	Clinical Decision Support Tool
ATEC	Autism Treatment Evaluation Checklist
NAC	N-AcetylCysteine
GABA	Gamma-Aminobutyric Acid (GABA)
OCD	Obsessive–Compulsive Disorder
OCPs	Oral Contraceptive Pills
eNOS	Endothelial Nitric Oxide Synthase
GSTO1	Glutathione S-Transferase Omega 1
GSTO2	Glutathione S-Transferase Omega 2
DRD2	Dopamine Receptor D2
HTR1A	5-Hydroxytryptamine Receptor 1A
HgA1c	Hemoglobin A1c
FOLR1	Folate Receptor Alpha
ESR2	Estrogen Receptor Beta
IL10	Interleukin 10
PPCDC	Phosphopantothenoylcysteine Decarboxylase
DBH	Dopamine Beta Hydroxylase
HTR1B	5-Hydroxytryptamine Receptor 1B
P5P	Pyridoxal 5′-Phosphate
HTR2A	5-Hydroxytryptamine Receptor 2A
CCL2	C-C Motif Chemokine Ligand 2
IL1B	Interleukin 1 Beta
CUBN	Cubilin
MTRR	Methionine Synthase Reductase
GSTP1	Glutathione S-Transferase Pi 1
